# Biomechanical comparison of different graft positions for single-bundle anterior cruciate ligament reconstruction

**DOI:** 10.1007/s00167-012-1951-4

**Published:** 2012-03-15

**Authors:** Yuki Kato, Akira Maeyama, Pisit Lertwanich, Joon Ho Wang, Sheila J. M. Ingham, Scott Kramer, Cesar Q. A. Martins, Patrick Smolinski, Freddie H. Fu

**Affiliations:** 1 Department of Orthopaedic Surgery, University of Pittsburgh, 3471 Fifth Avenue, 1010 Kaufmann Building, Pittsburgh, PA 15213 USA; 2 Department of Mechanical Engineering, University of Pittsburgh, Pittsburgh, PA USA; 3 Department of Orthopaedic Surgery, Nihon University, Tokyo, Japan

**Keywords:** Anterior cruciate ligament, Single-bundle, In situ force, Anterior tibial translation, Anatomic

## Abstract

**Purpose:**

Recent reports have highlighted the importance of an anatomic tunnel placement for anterior cruciate ligament (ACL) reconstruction. The purpose of this study was to compare the effect of different tunnel positions for single-bundle ACL reconstruction on knee biomechanics.

**Methods:**

Sixteen fresh-frozen cadaver knees were used. In one group (*n* = 8), the following techniques were used for knee surgery: (1) anteromedial (AM) bundle reconstruction (AM–AM), (2) posterolateral (PL) bundle reconstruction (PL–PL) and (3) conventional vertical single-bundle reconstruction (PL-high AM). In the other group (*n* = 8), anatomic mid-position single-bundle reconstruction (MID–MID) was performed. A robotic/universal force-moment sensor system was used to test the knees. An anterior load of 89 N was applied for anterior tibial translation (ATT) at 0°, 15°, 30° and 60° of knee flexion. Subsequently, a combined rotatory load (5 Nm internal rotation and 7 Nm valgus moment) was applied at 0°, 15°, 30° and 45° of knee flexion. The ATT and in situ forces during the application of the external loads were measured.

**Results:**

Compared with the intact ACL, all reconstructed knees had a higher ATT under anterior load at all flexion angles and a lower in situ force during the anterior load at 60° of knee flexion. In the case of combined rotatory loading, the highest ATT was achieved with PL-high AM; the in situ force was most closely restored with MIDMID, and the in situ force was the highest AM–AM at each knee flexion angle.

**Conclusion:**

Among the techniques, AM–AM afforded the highest in situ force and the least ATT.

## Introduction

Anterior cruciate ligament (ACL) reconstruction is one of the most common orthopaedic procedures performed in the United States, with approximately 105,000 surgeries performed per year [23]. The trans-tibial approach has been described as the recommended method for femoral tunnel placement [31]; however, this procedure poses the risk of a high/vertical placement of the femoral tunnel in the intercondylar notch and a discrepancy between the tunnel position and the point of ACL insertion [2]. The trans-tibial technique for single-bundle ACL reconstruction achieves good-to-excellent results in only 60 % of patients [6], and 20–30 % of athletes do not regain their previous level of performance [28]. To enhance its success rate, the conventional ACL reconstruction method needs to be further improved.

While double-bundle ACL reconstruction is gaining greater use, single-bundle reconstruction is still the most frequently performed method and is also useful when double-bundle reconstruction cannot be performed such as in cases with open growth plates, severe arthritic changes, multiple ligament injuries, a narrow notch, severe lateral femoral condyle bone bruising or tear of only one bundle [24]. The tibial ACL insertion site is a broad oval area, approximately 11 mm diameter in the coronal plane and 17 mm in the sagittal plane [1, 14]. Because of the fanning of the ligament, the tibial ACL insertion site is larger than the midsubstance and femoral attachment of the ligament [15]. With a single-bundle ACL reconstruction using a hamstrings graft, it is difficult to cover a large part of the ACL footprint with a round graft. Given this, little is known about the optimal tunnel position for anatomic single-bundle ACL reconstruction, although recent clinical studies recommend that the single-bundle graft be placed in the mid-bundle position of the ACL footprint [3].

To improve the single-bundle ACL reconstruction, while taking anatomic graft placement into account, the best tunnel positions must be evaluated. The purpose of this study was to compare the effect of different tunnel positions for single-bundle ACL reconstruction on knee biomechanics. It is hypothesized that the anatomic tunnel position is superior to the non-anatomic tunnel position and that the mid-bundle position is the most well balanced of the anatomic positions.

## Materials and methods

### Sample selection and grouping

**Fig. 1:**
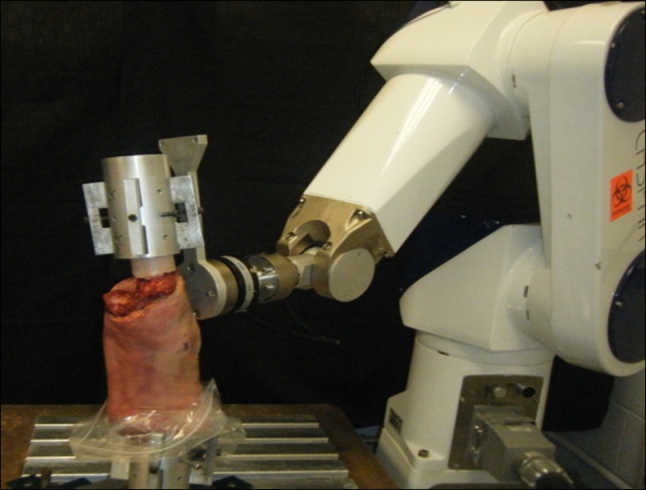
The robotic testing system. The tibia and femur were secured in aluminium cylinders using epoxy compound and placed in the testing system. The tibial cylinder was connected to the universal force-moment sensor

Sixteen fresh-frozen cadaveric knees were used in the study. Each specimen was screened by a CT scan and a manual examination, and the existence of an intact ACL was confirmed by arthroscopy. Knees were excluded if evidence of any of the following was present: ligament injuries, previous knee surgery, osteoarthritis and bony abnormalities. All specimens were stored at −20 °C and thawed at room temperature 24 h before the test. All soft tissues until a distance of approximately 10 cm away from both sides of the joint sides were removed. The exposed femur and tibia were secured in an epoxy compound (Bondo, Atlanta, Georgia, USA) for mounting in custom-made aluminium clamps. The femoral side was rigidly fixed to a base, while the tibial side was mounted to the end-effector of the robot through a universal force-moment sensor (UFS), as shown in Fig. 1. The specimens were kept moist throughout the testing.

**Fig. 2:**
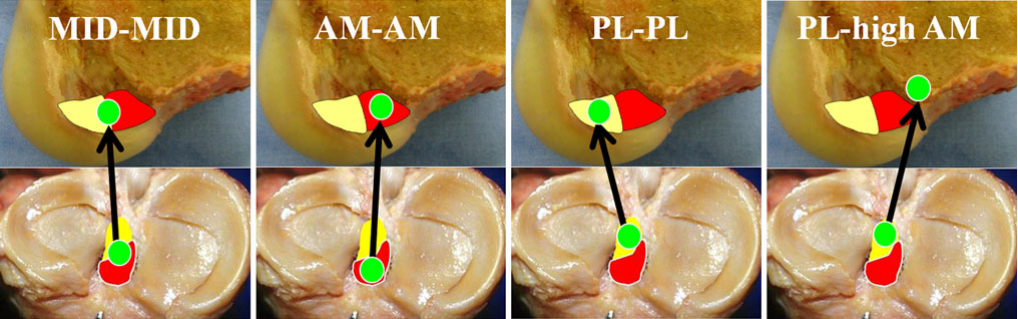
Four different tunnel positions for single-bundle anterior cruciate ligament reconstructions. Three anatomic anterior cruciate ligament reconstructions (MID–MID, AM–AM and PL–PL) and one non-anatomic anterior cruciate ligament reconstruction (PL-high AM) were compared

Four ACL reconstruction techniques were evaluated: (1) anatomic mid-bundle single-bundle reconstruction (MID–MID; *n* = 8), with the graft being placed from the midpoint between the tibial AM and PL footprints to the midpoint between the femoral AM and PL footprints; (2) anatomic AM bundle single-bundle reconstruction (AM–AM; *n* = 8), with the graft being placed from the tibial AM footprint to the femoral AM footprint; (3) anatomic PL bundle single-bundle reconstruction (PL–PL; *n* = 8), with the graft being placed from the tibial PL footprint to the femoral PL footprint; and (4) conventional single-bundle reconstruction (PL-high AM; *n* = 8), with the graft being placed from the tibial PL footprint to the femoral high AM position in the notch. Of the sixteen knees used, eight were used for MID–MID reconstructions, and the other eight knees were used for the remaining reconstructions (AM–AM, PL–PL and PL-high AM) with the three reconstructions being done in each knee (Fig. 2). There was no significant difference in the age or sex between the MID–MID reconstructed knee group and the multiple-reconstructed knee group (AM–AM, PL–PL and PL-high AM). The MID–MID reconstruction was performed in one group because it was difficult to place femoral tunnels at all positions (MID, PL, AM high AM) simultaneously; in particular, MID femoral tunnel creation between AM and PL femoral tunnels posed a risk of tunnel overlap.

### Interventions

**Fig. 3:**
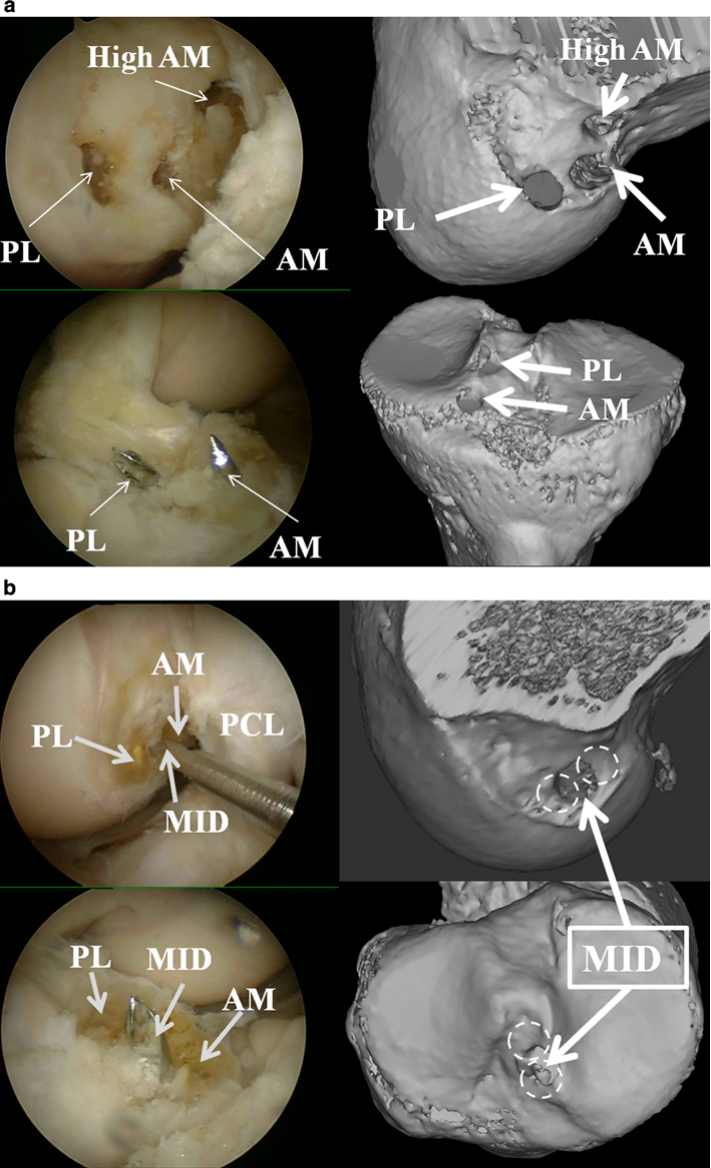
**a** Three femoral tunnels (PL, AM and high AM) and two tibial tunnels (PL and AM) were created in a multiple-reconstructed knee (arthroscopic view and 3D CT scan reconstruction). **b** For the MID–MID reconstruction, the tibial and femoral tunnels were drilled between the centre of the AM and PL footprints of the tibia and femur (arthroscopic view and 3D CT scan reconstruction)

A 3-portal technique was used with anterolateral, anteromedial and accessory medial portals with a 30° scope [13]. The two functional ACL bundles (AM and PL bundles) were differentiated by their tension patterns during knee motion. The ACL was removed with an electrothermal arthroscopy system (Vulcan, Smith and Nephew, Endoscopy, Andover, MA). After identifying the ACL foot print and removing the ACL, ACL reconstruction was performed. A guide wire (2.4 mm diameter) was inserted into the centre of each tibial ACL bundle footprint (AM and PL) from the anteromedial aspect of the tibia using a tibial drill guide system (Smith and Nephew Endoscopy, Andover, MA). The wire was then over drilled with a cannulated reamer (6 mm diameter). In 8 knees, three femoral tunnels (PL, AM and high AM) were created using a trans-portal technique [13]. A guide pin was inserted into the centre of the AM footprint, PL footprint and high AM position, which was located at the 11 or 1 o’clock position of the superoanterior portion of the ACL femoral footprint on the lateral wall of the intercondylar notch (Fig. 3a). The pin was then over drilled to the anterolateral femoral cortex by using a cannulated reamer (6 mm diameter). For the MID–MID reconstruction, the tibial and femoral tunnels were drilled between the centres of the AM and PL footprints of the tibia and femur in eight knees. Although one of the principles of an anatomic reconstruction is individualized surgery [28], 6 mm diameter bone tunnels were made for every reconstruction in this study to avoid AM and PL tunnel overlap (Fig. 3b). The knees were checked for the occurrence of tunnel overlap by CT scans after each test.

Previously harvested semitendinosus and gracilis tendons from human cadaver knees were used as grafts and were trimmed to a diameter of 6 mm. A No.5 braised polyester suture was whip stitched with a tapered needle into the free ends of the folded grafts. The graft was passed through the extra-cortical button loop (EndoButton, Smith and Nephew, Endoscopy, Andover, MA) to make it double stranded. At graft fixation, an initial tension of 60 N load was applied (ligament tension meter; Meira Corp, Nagoya, Aichi, Japan) [9] at 0° of knee flexion for the PL–PL reconstruction and at 30° of knee flexion for the other reconstructions (MID–MID, AM–AM and PL-high AM) [11, 16, 35]. Staples were used to fix the graft on the tibia.

### Outcome measurements

The robotic/UFS testing system was used to determine knee joint kinematics and in situ forces of the ligament and the reconstruction graft [27, 32, 33, 34]. The robotic manipulator (CASPAR, OrthoMaquet, Rastatt, Germany) is a six-joint, serial articulation device that allows 6-degree-of-freedom motion of the knee with repeatability of 0.02 mm at each joint according to the manufacturer. The UFS (model 4015; JR3 Inc, Woodland, California, USA) is capable of measuring 3 orthogonal forces and moments with repeatability of 0.2 N and 0.1 Nm, respectively, according to the manufacturer. A custom MATLAB program with a multitask operating system (Math Works Inc., Natick, Massachusetts, USA) was utilized to control the testing system, to monitor knee kinematics and calculate the in situ forces of the ACL and the reconstructed grafts. During the experiment, this testing system was operated in both the force- and displacement-control modes.

The specimen was initially mounted to the testing system at full extension of the knee (measured with a goniometer). The path of passive flexion–extension of the intact knee was determined with the robotic/UFS testing system by moving the tibia from full extension to 90° of flexion by 0.5° increments. At each incremental flexion of knee flexion, the forces and moments generated by the specimen in the remaining 5-degree-of-freedom were minimized by the iterative algorithm of the robot control software. The positions at full extension and 15°, 30° and 60° of flexion were used as the starting positions for applying external tibial loads throughout the test. The following external loading conditions were applied to the tibia: (1) 89 N of anterior tibial load (simulated KT 1000 test) [36] at full extension and 15°, 30° and 60° of flexion and (2) a combined 7 Nm valgus torque and 5 Nm internal tibial rotation torque (simulated pivot-shift test) [20] at 0°, 15°, 30° and 45° of flexion. The anterior tibial load was as applied since the ACL is a major restraint to anterior tibial translation, and its application corresponds to the Lachman test and anterior drawer test. The force of 89 N is equivalent to that used in the KT-1000 arthrometer [10]. The combined valgus and internal tibial torque was chosen to simulate the pivot-shift test. While the external tibial loads were applied at each flexion angle, the 5-degree-of-freedom kinematics, forces, and moments of the intact knee were monitored. The ACL was transected arthroscopically, and the knee was tested with both modes of the testing system. Initially, the same loading conditions were employed in force control mode of the testing system to obtain kinematics of the ACL-deficient knee at each testing angle. Subsequently, in displacement-control mode, the identical path of motion of the intact knee was repeated in the ACL-deficient knee while new forces and moments of the specimen were recorded. By the principle of superposition, the vectorial difference in the measured forces between the intact and the ACL-deficient knee during the same path of motion gives the in situ force of the ACL [27, 33, 34].

All ACL reconstructions were performed sequentially. For the multiply reconstructed knees, the three ACL reconstructions, AM–AM, PL–PL and PL-high AM, were performed in random order. For the single reconstruction knees, only anatomic mid-bundle single-bundle reconstruction (MID–MID) was performed. The same graft (hamstring) and the same tibial fixation technique were used in all the reconstructions. External tibial loads were applied, and the kinematics of each reconstructed knee was analysed. After graft removal, the path of motion of the reconstructed knee at each testing angle was replayed while new forces and moments were monitored. The in situ force of the graft with each reconstruction technique was the vector difference in the measured forces between the reconstructed knee and the ACL-deficient knee with an identical path of motion.

### Statistical analysis

Differences in ATT and in situ force at the different flexion angles were analysed using the Kruskal–Wallis test for comparison of all groups and the Mann–Whitney *U* test between all the pairwise comparisons. A Bonferroni approach was used to adjust the alpha level for the pairwise post hoc comparisons, and statistical significance was assumed when *p* < 0.05 for the Kruskal–Wallis test and *p* < 0.01 for the Mann–Whitney *U* test. Statistical analysis was performed using the software package SPSS version 17.0 (SPSS Inc., Chicago, IL, USA). Pairwise comparisons were made between the data of all reconstruction methods and the intact ACL and between the AM–AM, PL–PL and MID–MID reconstructions and the PL-high AM reconstruction.

## Results

### Anterior tibial translation during anterior load

**Fig. 4:**
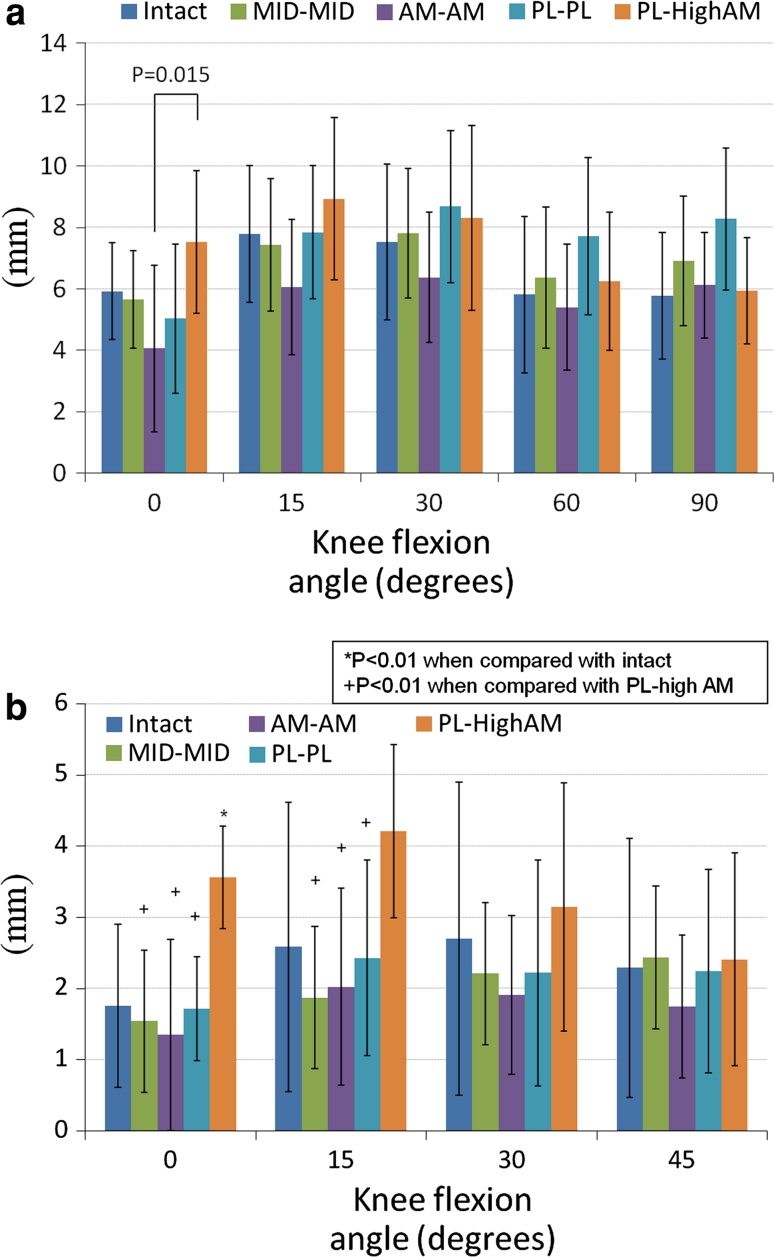
**a** Anterior tibial translation (ATT) (mm) in response to the anterior tibial load. **b** Coupled anterior tibial translation (ATT) (mm) in response to the combined rotatory load

The PL-high AM reconstructed knee had the largest anterior displacement with the anterior tibial load when compared with the other reconstructed knees at 0° and 15° of knee flexion (Fig. 4a). At 30° and 60° of knee flexion, the PL–PL reconstructed knee had the largest displacement. However, overall there was no significant difference in ATT between the intact knees and the different reconstructions.

### Coupled anterior tibial translation during the combined rotatory load

The PL-high AM reconstructed knee had a greater anterior displacement during the combined rotatory load than the intact knee at 0°, 15° and 30° of knee flexion, although this difference was significant only at 0° of knee flexion (*p* = 0.001) (Fig. 4b). The PL-high AM reconstructed knee had a significantly larger ATT at 0° and 15° of flexion under combined rotatory loading than the MID–MID (*p* = 0.003–0°, 0.007–15°), AM–AM (*p* = 0.005–0°, 0.005–15°) and PL–PL (*p* = 0.001–0°, 0.01–15°) reconstructions.

### In situ forces during the anterior load

**Fig. 5:**
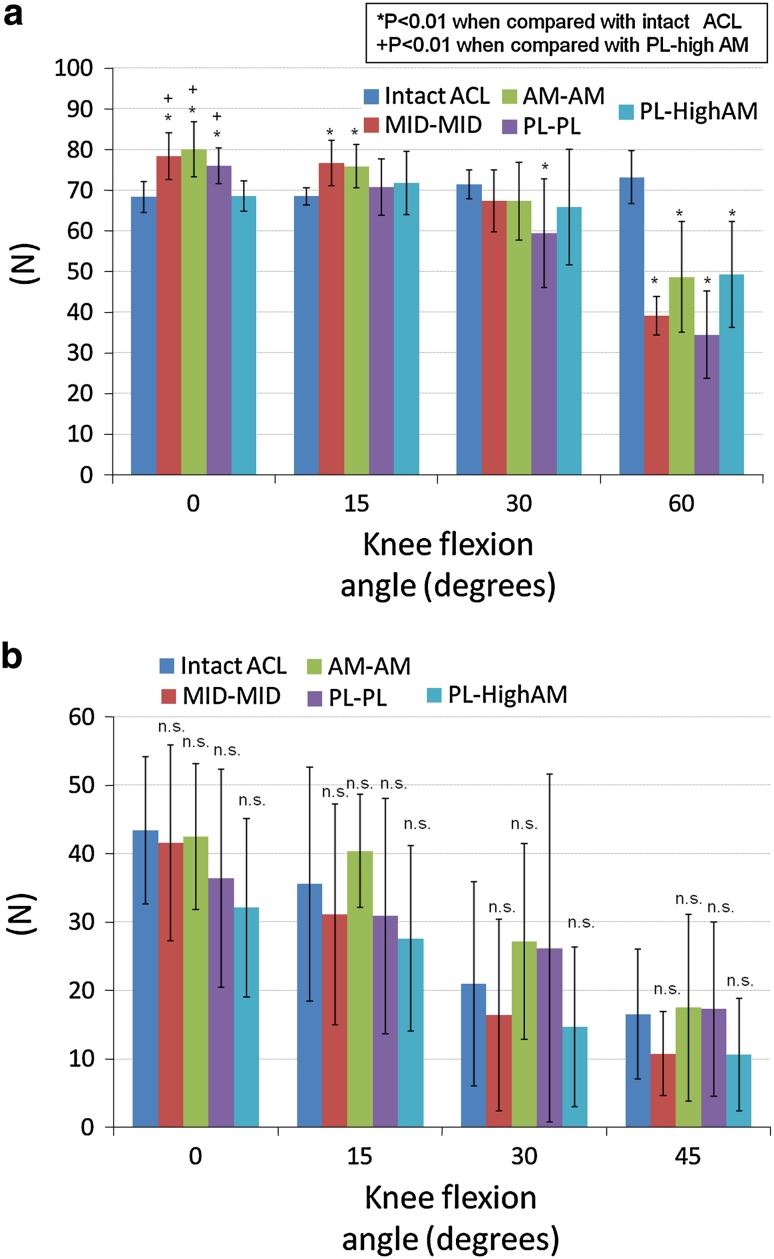
**a** In situ forces (N) in response to the anterior tibial load. **b** In situ forces (N) in response to the combined rotatory load

The MID–MID, AM–AM and PL–PL grafts had significantly higher in situ forces in response to the anterior load than the intact ACL at 0° of knee flexion (*p* = 0.000, 0.000 and 0.001, respectively). The PL-high AM graft had a significantly lower in situ force at 0° of knee flexion as compared to the MID–MID, AM–AM and PL–PL grafts (*p* = 0.005, 0.001 and 0.005, respectively) (Fig. 5a). The MID–MID and AM–AM grafts had significantly higher in situ forces than the intact ACL at 15° of knee flexion (*p* = 0.002 and 0.002, respectively). The PL–PL graft had a significantly lower in situ force than the intact ACL at 30° and 60° of knee flexion (*p* = 0.003 and 0.000, respectively). At 60° of knee flexion, all grafts had a significantly lower in situ force when compared with the intact ACL (*p* = 0.000).

### In situ forces during the combined rotatory load

The reconstructed grafts showed no significant difference from the intact ACL with respect to the in situ force under the combined rotatory loading (Fig. 5b). The PL-high AM had the lowest in situ force at 0°, 15°, 30° and 45° of knee flexion, whereas the AM–AM graft had the highest in situ force at 0°, 15°, 30° and 45° of knee flexion.

## Discussion

In this study, different graft positions for single-bundle ACL reconstruction were compared to identify the position that best restores intact knee kinematics. The results show that anatomic reconstruction restores normal knee kinematics better than the non-anatomic ACL reconstruction.

The findings of graft isometry studies have supported femoral tunnel placement at a high position in the intercondylar notch in a non-anatomic location [8, 37], and often, the tibial tunnel must be placed slightly posteriorly to avoid roof impingement [17]. On the basis of these recommendations, the tibial tunnel is placed within the PL bundle of the ACL footprint [36]. Nevertheless, conventional single-bundle ACL reconstruction has consistently yielded high stability rates, high patient satisfaction and low revision rates, in the hands of experienced surgeons [21]. However, placement of the femoral tunnel lower on the clock face of the notch has been attracting growing attention [18, 22, 26], which indicates that the anatomic placement of the femoral tunnel position is gaining favour. In a biomechanical cadaveric study, Loh et al. [22] found that grafts in the 10 o’clock position afforded better resistance to rotatory loads than grafts in the 11 o’clock position. The present study has shown that the conventional vertical reconstructed knee (PL-high AM) had a large ATT in response to an anterior load and the combined rotatory loads at 0°, 15° and 30° of knee flexion, although the values were not statistically significant in all cases.

Steiner et al. [29] recommended that the tibial tunnel should be placed anterior and medial to the footprint, as long as it does not cause graft impingement [17]. The tibial tunnel positions of the AM–AM and MID–MID reconstructions are more anterior than that of the conventional (PL-high AM) and PL–PL reconstructions. Although these tunnels are placed more anteriorly and could potentially cause impingement [19], the presence of the lower femoral tunnel was expected to reduce this possibility [28].

Recent studies have shown that the optimal femoral and tibial insertion sites for an ACL graft are within the anatomic footprint of the ACL [5, 12]. Some studies have shown that conventional single-bundle ACL reconstruction may be mechanically inferior to the native ACL position [7, 22, 25], which suggests that the anatomic positioning of a single-bundle ACL reconstruction may provide better control of knee stability than non-anatomic positioning.

A recent clinical study demonstrated that placement of a posterior femoral tunnel and an anterior tibial tunnel afforded good results [25]. In the present study, the AM–AM graft yielded a high in situ force in response to the external loads at all knee flexion angles. However, there is some concern about the increased re-injury rate because of the large strain in the AM–AM reconstructed graft. It should be noted that with all methods the graft had a lower in situ force than the intact ligament during ATT loading at higher flexion angles (60°).

The AM and PL bundles have a synergistic relationship [7], and the native ACL bundles do not function independently. The AM–AM reconstruction yielded better stability than any of the other reconstructions, and the MID–MID reconstruction did not have any major weaknesses. Steiner [29] recommended that the femoral tunnel be placed at the centre of the femoral footprint, although he recommended that the tibial tunnel be placed at the AM footprint.

Recent biomechanical studies have emphasized the importance of the PL bundle for constraining rotatory instability [36]. Contrary to previous findings, the results of the current study showed that the displacement of the AM–AM reconstructed knee during the anterior tibial and the combined rotatory loading at 0°, 15°, 30° and 45° of knee flexion was less than that of the PL–PL reconstructed knee. However, the PL–PL reconstructed knee had less force than during the combined application of external loads at 0°, 15° and 30° of knee flexion. These results suggest that the AM graft can play a role in rotatory stability in addition to the PL graft; however, the PL–PL reconstructed knee tended to have a higher ATT under anterior loading conditions at over 30° of knee flexion, suggesting that the PL–PL tunnel position is not suitable for a single-bundle reconstruction.

This study has some limitations. The external loads applied in the present study were lower than those used in previous biomechanical studies [30, 33, 36]. In spite of this, it is believed that the controlled experimental study shows the same trends. The study used 6-mm-diameter grafts, whereas 9 mm or larger diameter grafts are used clinically in single-bundle ACL reconstructions. While the graft diameter does affect graft stiffness, it can be noted that the smaller grafts did restore the intact anterior tibial translation and in some cases the in situ force of the graft was greater than that of the intact ACL. Moreover, a recent study comparing 6- and 9-mm tunnels for a single-bundle ACL reconstruction revealed that increasing the graft size did not improve the time-zero biomechanical stability [4]. Grafts were made from both semitendinosus and gracilis tendons and there may be a difference in these tissues. In the present study, the femoral high AM tunnel, which is usually created using a trans-tibial technique, was created using a trans-portal technique, in order to avoid damage to the other the tibial tunnels. All the knee motions evaluated by the robotic system were static and at time zero, so the influence of healing could not be assessed. A sample-size analysis or post hoc power analysis was not performed and, despite the fact that statistically significant results were obtained, the large number of variables evaluated could increase the type 2 error. Although it cannot be claimed that the results of this study apply directly to single-bundle ACL reconstructions with larger grafts, it does emphasize the importance of anatomical tunnel position in knee biomechanics.

## Conclusion

The in situ force and stability of the intact ACL was most closely reproduced by AM–AM single-bundle ACL reconstruction technique, as compared to the other ACL reconstruction techniques tested.
